# Analysis of the 3D distribution of stacked self-assembled quantum dots by electron tomography

**DOI:** 10.1186/1556-276X-7-681

**Published:** 2012-12-18

**Authors:** Jesús Hernández-Saz, Miriam Herrera, Diego Alonso-Álvarez, Sergio I Molina

**Affiliations:** 1 INNANOMAT Group, Departamento de Ciencia de los Materiales e I.M. y Q.I., Facultad de Ciencias, Universidad de Cádiz, Campus Río San Pedro, s/n, Puerto Real, Cadiz, 11510, Spain; 2 Instituto de Microelectrónica de Madrid, CNM (CSIC), c/Isaac Newton 8, PTM, Tres Cantos, Madrid, 28760, Spain

**Keywords:** Focused ion beam, Electron tomography, Needle-shaped samples, Quantum dots, Semiconductor, transmission electron microscopy, High angle annular dark field, 81.05.Ea, 81.07.Ta, 68.37.Ma

## Abstract

The 3D distribution of self-assembled stacked quantum dots (QDs) is a key parameter to obtain the highest performance in a variety of optoelectronic devices. In this work, we have measured this distribution in 3D using a combined procedure of needle-shaped specimen preparation and electron tomography. We show that conventional 2D measurements of the distribution of QDs are not reliable, and only 3D analysis allows an accurate correlation between the growth design and the structural characteristics.

## Background

Most optoelectronic devices based in quantum dots (QDs) such as optical amplifiers [1], infrared detectors [2], or lasers [3] require stacking of multiple QDs layers to enhance properties as the number of photons emitted or absorbed per unit area. For small spacer layers, QDs tend to align vertically because of the strain fields caused by the buried dots [4,5]. These strain fields have a strong effect in the size and shape of the QDs and consequently, in the optoelectronic properties of the corresponding devices [6-11]. The vertical distribution of the QDs has a direct effect in its electronic structure due to a possible electron tunneling between layers [12], and it has also been found to influence optical properties such as the photoluminescence emission of the structure [13]. Because of this, understanding the 3D distribution of stacked QDs is essential to understand and optimize the functional properties of a wide range of devices.

Although various techniques have been used to assess the vertical distribution of QDs [14-16], one of the most powerful techniques for this purpose is transmission electron microscopy (TEM) because it gives direct evidence of the location of the QDs. However, the vertical alignment of the stacking of QDs is often analyzed by TEM from 2D projections of the volume of the sample in one or several directions [17,18], losing 3D information and therefore, making the complete correlation with the optical characteristics unfeasible. To solve this problem, electron tomography is the most appropriate technique. An accurate 3D reconstruction in electron tomography needs the accomplishment of some requirements, the most important one being that the input 2D images must be the true projections of the original 3D object [19]. This condition can be met by using high-angle annular dark field (HAADF) scanning transmission electron microscopy (STEM) images for the tilting series, given that the diffraction effects present in conventional bright field TEM images are minimized.

On the other hand and regarding the specimen, it is required that the electron beam crosses a constant thickness of the electron-transparent foil when traveling through the sample during the tilting series. This is not accomplished by the thin foils prepared by the conventional method of specimen preparation, and only cylindrical or conical-shaped specimens with the symmetry axis parallel to the tilting axis would meet this requirement. The fabrication of these specimens in the form of needles has been recently accomplished with the use of a dual beam scanning electron microscopy-focused ion beam instrument (FIB), and it has been applied to atom probe analyses [20], electron tomography studies [21], and 3D-STEM observations [22].

In this paper, we have analyzed the vertical alignment of InAs/GaAs stacked QDs grown between GaP strain compensation layers by electron tomography with HAADF images, using a needle-shaped specimen fabricated by FIB. Contrary to what is derived from a 2D conventional analysis, we have observed a considerable deviation of the vertical stacking from the growth direction, which is a key finding for the future interpretation of its functional properties.

## Methods

The sample studied in this work consists of a stack of 50 layers of self-assembled InAs QDs grown by molecular beam epitaxy at 510°C on GaAs (001). For each layer, 1 ML of GaP have been deposited 1.53 nm below and 12.6 nm above the InAs layer (2 ML of InAs) in order to compensate the strain. Further details about the growth of this sample are included in Alonso-Alvarez et al. [12]. FIB sample preparation has been carried out using a dual-beam FEI Quanta200 3D FIB (FEI Company, Eindhoven, Netherlands) instrument equipped with an *in situ* Omniprobe micromanipulator (Dallas, TX, USA), where the ion acceleration voltage ranges from 5 to 30 kV.

Sixty-one HAADF-STEM images have been obtained over an angular range of 120° with a tilting step of 2° in a JEOL JEM 2010F electron microscope (JEOL Ltd., Tokyo, Japan) with a field emission gun working at 200 kV using a Fischione tomography holder (model 2030) (Fischione Instruments, 9003 Corporate Circle Export, PA, USA). The tilt series has been accurately aligned using the Inspect 3D software of FEI Company with the cross-correlation method in combination with the least-squares alignment mode with the AMIRA software (Amira, Merignac Cedex, France). The 3D reconstruction has been carried out using the simultaneous iterative reconstruction technique and is visualized with the software AMIRA. Because of the high contrast of the InAs QDs in the HAADF-STEM images, manual segmentation of the tomogram was carried out in order to locate the QDs. The position of the QDs has been considered as the geometric center of the QDs in the tomogram.

### FIB sample preparation method

Needle-shaped specimens fabricated for electron tomography need to meet specific requirements, often more strictly than for other applications as atom probe tomography, such as reduced needle diameter and minimized surface amorphous layer. We have previously reported in detail the procedure to fabricate such needles from semiconductor materials [23]. In short, the method consists on protecting the surface of the bulk material by depositing a Pt layer, followed by milling a 1- to 2-μm-thick lamella using the *in situ* lift-out method [24] and then sculpting a needle using annular patterns of variable diameter. In Hernández-Saz et al. [23], the sample consisted of one layer of InAs QDs grown on InP. However, in the present study, the sample consists of a larger number of InAs QDs layers (50) and grown on a different substrate (GaAs). The fabrication of needles from this sample requires some modifications in the preparation method in order to optimize the structural characteristics of the specimen, which are explained below.

With regard to the substrate, GaAs has a lower sputter yield than InP [25] which means that for a given ion beam current and voltage, the time required for a specific milling step will be higher. In this case, attention should be paid to a possible spatial drift of the sample with time, as its effects on the final geometry of the specimen will be more pronounced.

Regarding the higher number of QDs layers in the structure, care should be taken to sculpt a needle with reduced diameter along a larger distance in the needle axis in order to include all the QDs layers, about 900 nm in this sample. In soft materials such as III-V semiconductors, milling a needle with the ion beam following an annular pattern normally produces a typical conical shape where the diameter increases rapidly as the distance from the top of the needle is raised. To avoid this, an increase in the annular milling steps has been introduced in the procedure, which also helps avoiding the effect of the drift mentioned before. Table 1 shows the steps followed for milling a needle from a GaAs lamella. As it can be observed, the inner diameter is reduced slowly, in a number of steps, in order to obtain a needle with a nearly cylindrical shape. The annulus shape of the pattern is etched from the external surface of the needle inwards with depth of 500 nm and dwell time of 1 μs.

**Table 1:** Parameters used in each step of the annular milling process to fabricate GaAs needles with a reduced diameter along a large range

**Step**	**Inner diameter (nm)**	**Outer diameter (nm)**	**Current (pA)**	**Voltage (kV)**
1	1,000	1,500	100	30
2	800	1,400	81	20
3	700	1,200	23	20
4	600	1,000	23	20
5	500	850	23	20
6	400	700	4	20
7	300	600	4	20
8	150	400	4	20
9	-	-	70	5

The last step is to clean the amorphous layer around the needle.

## Results and discussion

**Figure 1:**
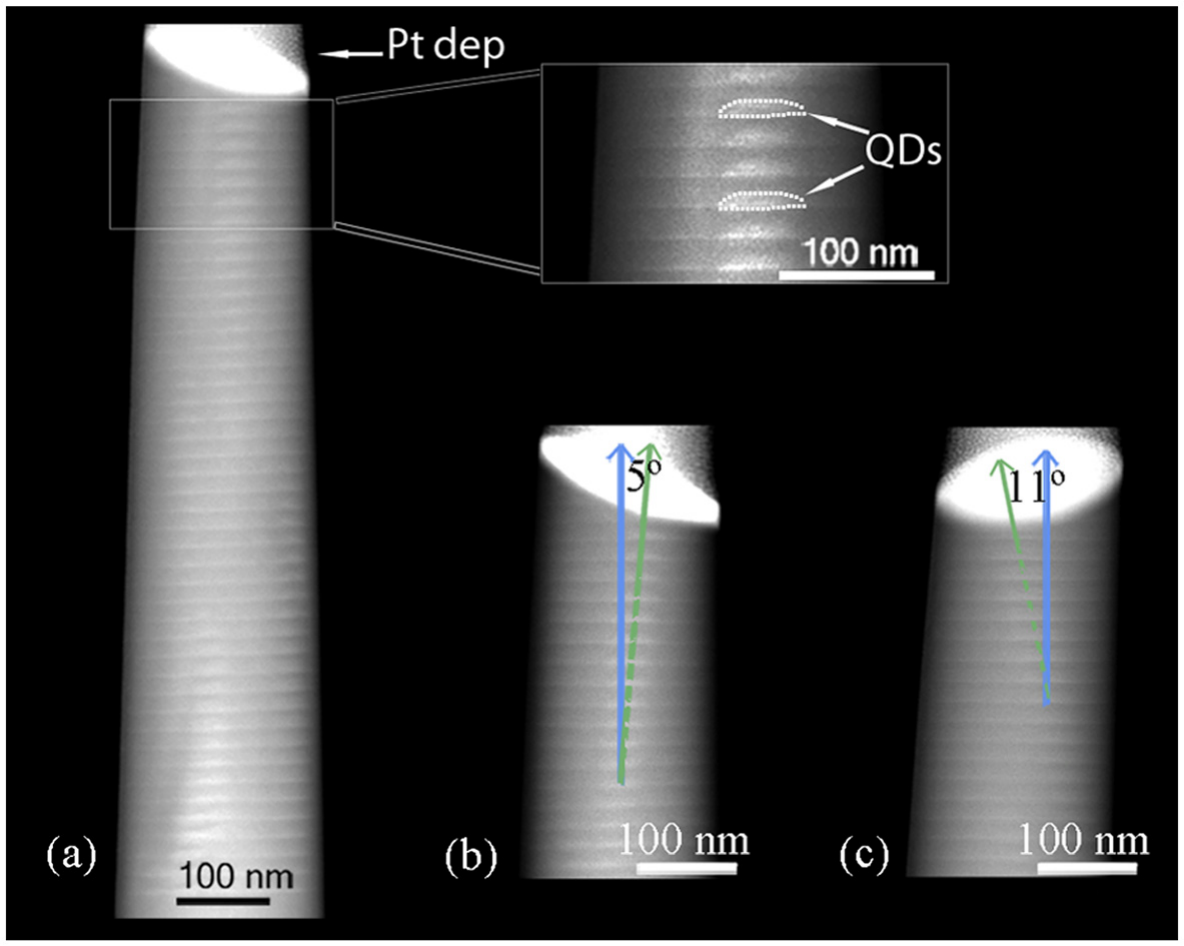
**Cross-sectional HAADF images of the needle-shaped specimen taken at different rotation angles.** Note that the angles between the stacking of QDs and the growth direction are different for the three images: (**a**) 0°, (**b**) 5°, and (**c**) 11°.

Figure 1 (a) shows a HAADF image of a specimen prepared by FIB following the procedure described above. As it can be observed, the needle has a shape close to cylindrical and its diameter is small enough so that the different QDs layers are visible, showing that the proposed fabrication method was successful.

In this image, the InAs QDs can be clearly observed as they exhibit brighter contrast than the GaAs matrix because of the higher average *Z* number. However, in HAADF images, the static atomic displacements of the atoms, because of the strain in the epitaxial layers, also play an important role in the observed contrast [26,27]. Because of the rounded shape of the QDs, they are not expected to show sharp upper interfaces when observed by HAADF but with diffused boundaries, in which the contrast is gradually reduced at the edge, as it is shown in the image. Regarding the vertical stacking of the QDs, it is worth mentioning that we have not found a stacking running across all the 50 layers as expected, but only up to approximately 12 to 15 QDs. This could be detrimental to the functional properties of this structure, and it is a consequence of the strain fields in the structure.

About the vertical alignment of the QDs, from the micrograph in the inset of Figure 1 (a) it seems to be parallel to the growth direction. In many cases, this is the expected distribution of the QDs since the non-perfect alignment of the QDs has been reported to influence the electron wavefunction [28] and to reduce the exchange energy between electronic states [29]. However, it should be highlighted that TEM cross section images are 2D projections of the sample and therefore, the volume information is lost; this should be taken into account to avoid the misinterpretation of the images. In this regard, (b) and (c) in Figure 1 show HAADF images of the same needle-shaped specimen as in (a) in Figure 1 but taken at different rotation angles, 90° apart from each other, and −10° and 80° from the micrograph in (a) in Figure 1, respectively. The unusual geometry of the needle-shaped specimen fabricated by FIB in this study allowed us to obtain a higher number of projections than possible from the conventional thin foils, providing interesting additional information of the sample. As it can be observed, at these rotation angles, the stacking of QDs is not vertically aligned anymore. Instead, deviation angles of 5° and 11° with respect to the growth direction have been measured. Other values for the vertical alignment of the QDs have been measured from different rotation angles. These experimental results to evidence that the conclusions obtained from the conventional 2D analysis of the stacking of QDs often found in the literature are not reliable and would mislead the interpretation of the functional properties of these nanostructures, being the 3D analysis of the sample as an essential step.

**Figure 2:**
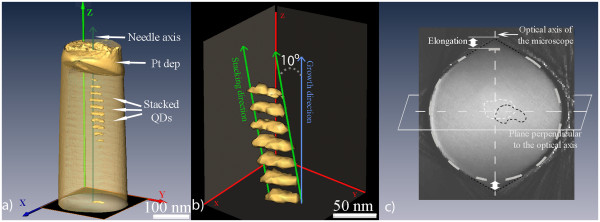
**The surfaces render of the reconstructed volume and an axial slice through the needle.** (**a**) Semi-transparent external surface of the tomogram of the needle with opaque surfaces for the QDs below the platinum deposition. A projection of one of the central QDs is included in the base of the needle. (**b**) Segmentation of the QDs in the tomogram, showing that the stacking of QDs follows a straight line that deviates 10° from the growth direction. (**c**) Slice through the upper QD of the reconstructed tomogram where we have superimposed a circle to evidence the elongation in the direction of the optical axis of the microscope. The upper and lower QDs of the Figure 2b have been included with a white and black dotted line respectively.

In order to obtain 3D information from the sample, we have acquired a tilt series of HAADF images, and we have computed the tomogram using these images. The results are shown in Figure 2a,b. Figure 2a shows a general view of the needle, including the upper stacking of QDs and the platinum deposition. For the analysis of the distribution of the QDs, a segmentation of the reconstructed structure was carried out, as shown in Figure 2b. This figure reveals that the real distribution of the QDs consist of a stacking that follows a straight line that deviates 10° from the growth direction Z, which is quite different from the results obtained from Figure 1a. From this analysis, we have also observed that there is an asymmetry in the size of the QDs, being around 30% smaller in one direction than in the perpendicular one in the growth plane.

It is worth mentioning that often the 3D information obtained from tomography analyses suffers from the missing wedge artifact due to a lack of information for high rotation angles. This causes an elongation of the features in the sample along the microscope optical axis (in our case, parallel to the wetting layers). Figure 2c shows an axial slice through the reconstructed needle, where this elongation is observed. We have superimposed a circle along the surface of the needle to evidence this elongation more clearly. From this figure, we have calculated an elongation percentage due to the missing wedge of 1.14%. We have measured the vertical alignment of the dots using the location of the center of each dot and because of the calculated elongation, this position will be displaced from its real location. The maximum error in the location of the QDs would occur for dots placed close to the surface of the needle, and where the QDs alignment has a component parallel to the optical axis of the microscope. In this case, the error in the angle between the QDs vertical alignment and the growth direction would be of 3.5°. This error could be minimized using needle-shaped specimens in combination with last generation tomography holders that allow a full tilting range. On the other hand, for QDs stacking included in a plane perpendicular to the microscope optical axis located in the center of the needle (as shown in Figure 2c), there would be no error in the measurement of the angle. In our case, the vertical alignment of the dots is closer to this second case. In Figure 2c we have included the position of the upper QD in the stacking with a white dotted line, and of the lower QD with a black dotted line. As it can be observed, both dots are very close to the center of the needle, and the vertical alignment forms an angle close to 90° with the optical axis; therefore, the error in the measurement of the QDs vertical alignment is near to 1°.

The observed deviation from the growth direction of the stacking of QDs is caused by the elastic interactions with the buried dots and by chemical composition fluctuations [16,30]. However, other parameters such as the specific shape of the QDs [4,5,31], elastic anisotropy of the material [4,5,30,31], or the spacer layer thickness [4,5,30] need to be considered as well to predict the vertical distribution of the QDs. Understanding these complex systems needs both a strong theoretical model and precise experimental measurements to compare the obtained results. Our work provides these experimental data. The correlation of these results with the growth design and with the functional properties of these structures will allow closing the loop to optimize the performance of devices based in stacking of QDs.

## Conclusions

In summary, we have analyzed the 3D distribution of InAs/GaP/GaAs stacked QDs by electron tomography using HAADF images. For this, we have optimized the needle-shaped specimen fabrication procedure by FIB for samples with multiple layers of QDs. We have found that contrary to what could be derived from a 2D conventional TEM analysis, the QDs do not follow a vertical alignment, but there is a deviation angle of 10° ± 1°. The unambiguous determination of the 3D distribution of QDs is a key for the interpretation of the optoelectronic properties of devices based in stacking of QDs.

## Abbreviations

FIB: Focused ion beam; GaAs: Gallium arsenide; HAADF: High angle annular dark field; InAs: Indium arsenide; InP: Indium phosphorus; QDs: Quantum dots; STEM: Scanning transmission electron microscopy; TEM: Transmission electron microscopy.

## Competing interests

The authors declare that they have no competing interests.

## Authors’ contributions

JHS has participated in the design of the study; prepared the experimental specimens, carried out the STEM images, the alignment, and the reconstruction of the data; taken part in discussions and in the interpretation of the result; and written the manuscript. MH has designed the study, participated in the acquisition of the STEM images, performed data analysis; she has supervised the research and revised the manuscript and has taken part in discussions and in the interpretation of the results. DAA has grown the samples and has taken part in discussions and in the interpretation of the results. SIM has conceived the study, participated in its design, supervised the writing of the manuscript and the experimental part. All the authors have read and approved the final manuscript.

## Authors’ information

JHS is a PhD student at the Universidad de Cádiz. MH is an associate professor at the Departamento de Ciencia de los Materiales e Ingeniería Metalúrgica y Química Inorgánica, Universidad de Cádiz. DAA holds a postdoctoral position as Research Associate in the School of Engineering and Physical Sciences at Heriot-Watt University and the Scottish Institute for Solar Energy Research (SISER). SIM is a full professor at the Departamento de Ciencia de los Materiales e Ingeniería Metalúrgica y Química Inorgánica, Universidad de Cádiz.

## References

[B1] Wegert M Majer N Ludge K Dommers-Volkel S Gomis-Bresco J Knorr A Woggon U Scholl E Nonlinear gain dynamics of quantum dot optical amplifiers Semicond Sci Technol 2011 26 014008 10.1088/0268-1242/26/1/014008

[B2] Bhattacharya P Mi Z Yang J Basu D Saha D Quantum dot lasers: from promise to high-performance devices J Cryst Growth 2009 311 1625 1631 10.1016/j.jcrysgro.2008.09.035

[B3] Gong Q Chen P Li SG Lao YF Cao CF Xu CF Zhang YG Feng SL Ma CH Wang HL Quantum dot lasers grown by gas source molecular-beam epitaxy J Cryst Growth 2011 323 450 453 10.1016/j.jcrysgro.2010.12.014

[B4] Tersoff J Teichert C Lagally MG Self-organization in growth of quantum dot superlattices Phys Rev Lett 1996 76 1675 1678 10.1103/PhysRevLett.76.1675 10060489

[B5] Wang ZM Holmes K Mazur Yu I Salamo GJ Fabrication of (In, Ga)As quantum-dot chains on GaAs(100) Appl Phys Lett 2004 84 1931 1933 10.1063/1.1669064

[B6] Wang Zh M Seydmohamadi S Lee JH Salamo GJ Surface ordering of (In, Ga)As quantum dots controlled by GaAs substrate indexes Appl Phys Lett 2004 85 5031 5033 10.1063/1.1823590

[B7] Zhi D Davock H Murray R Roberts C Jones TS Pashley DW Goodhew PJ Joyce BA Quantitative compositional analysis of InAs/GaAs quantum dots by scanning transmission electron microscopy J Appl Phys 2001 89 2079 2083 10.1063/1.1337921

[B8] Barker JA O’Reilly EP Theoretical analysis of electron–hole alignment in InAs-GaAs quantum dots Physical Review B 2000 61 13840 13851 10.1103/PhysRevB.61.13840 11017359

[B9] Rosenauer A Oberst W Litvinov D Gerthsen D Forster A Schmidt R Structural and chemical investigation of In(0.6)Ga(0.3)As Stranski-Krastanow layers buried in GaAs by transmission electron microscopy Physical Review B 2000 61 8276 8288 10.1103/PhysRevB.61.8276

[B10] Fry PW Itskevich IE Mowbray DJ Skolnick MS Finley JJ Barker JA O’Reilly EP Wilson LR Larkin IA Maksym PA Hopkinson M Al-Khafaji M David JPR Cullis AG Hill G Clark JC Inverted electron–hole alignment in InAs-GaAs self-assembled quantum dots Phys Rev Lett 2000 84 733 736 10.1103/PhysRevLett.84.733 11017359

[B11] Nuntawong N Tatebayashi J Wong PS Huffaker DL Localized strain reduction in strain-compensated InAs/GaAs stacked quantum dot structures Appl Phys Lett 2007 90 163121 10.1063/1.2730732

[B12] Alonso-Alvarez D Taboada AG Ripalda JM Alen B Gonzalez Y Gonzalez L Garcia JM Briones F Marti A Luque A Sánchez AM Molina SI Carrier recombination effects in strain compensated quantum dot stacks embedded in solar cells Appl Phys Lett 2008 93 123114 10.1063/1.2978243

[B13] Jin-Phillipp NY Phillipp F Strain distribution in self-assembled InP/GaInP quantum dots J Appl Phys 2000 88 710 715 10.1063/1.373726

[B14] Srinivasan T Singh SN Tiwari U Sharma RK Muralidharan R Rao DVS Balamuralikrishnan R Muraleedharan K Structural and photoluminescence characteristics of molecular beam epitaxy-grown vertically aligned In0.33Ga0.67As/GaAs quantum dots J Cryst Growth 2005 280 378 384 10.1016/j.jcrysgro.2005.04.010

[B15] Ouattara L Ulloa JM Mikkelsen A Lundgren E Koenraad PM Borgstrom M Samuelson L Seifert W Correlation lengths in stacked InAs quantum dot systems studied by cross-sectional scanning tunnelling microscopy Nanotechnology 2007 18 145403 10.1088/0957-4484/18/14/145403

[B16] Molina SI Ben T Sales DL Pizarro J Galindo PL Varela M Pennycook SJ Fuster D Gonzalez Y Gonzalez L Determination of the strain generated in InAs/InP quantum wires: prediction of nucleation sites Nanotechnology 2006 17 5652 5658 10.1088/0957-4484/17/22/020 21727338

[B17] Shoji Y Oshima R Takata A Okada Y The effect of spacer layer thickness on vertical alignment of InGaAs/GaNAs quantum dots grown on GaAs(3 1 1)B substrate Physica E 2010 42 2768 2771 10.1016/j.physe.2009.11.095

[B18] Gutierrez M Herrera M Gonzalez D Garcia R Hopkinson M Role of elastic anisotropy in the vertical alignment of In(Ga)As quantum dot superlattices Appl Phys Lett 2006 88 193118 10.1063/1.2202190

[B19] Radon J Ueber die Bestimmung von Funktionen durch ihre integralwerte laengs gewisser Mannigfaltigkeiten Math-Phys Kl 1917 69 262 277

[B20] Lozano-Perez S A guide on FIB preparation of samples containing stress corrosion crack tips for TEM and atom-probe analysis Micron 2008 39 320 328 10.1016/j.micron.2007.12.003 18258443

[B21] Ke XX Bals S Cott D Hantschel T Bender H Van Tendeloo G Three-dimensional analysis of carbon nanotube networks in interconnects by electron tomography without missing wedge artifacts Microsc Microanal 2010 16 210 217 20187989 10.1017/S1431927609991371

[B22] Ozasa K Aoyagi Y Iwaki M Hara M Maeda M Nanofabrication of cylindrical STEM specimen of InGaAs/GaAs quantum dots for 3D-STEM observation Ultramicroscopy 2004 101 55 61 10.1016/j.ultramic.2004.04.001 15450652

[B23] Hernandez-Saz J Herrera M Molina SI A methodology for the fabrication by FIB of needle-shape specimens around sub-surface features at the nanometre scale Micron 2012 43 643 650 10.1016/j.micron.2011.11.011

[B24] Langford RM Rogers M In situ lift-out: steps to improve yield and a comparison with other FIB TEM sample preparation techniques Micron 2008 39 1325 1330 10.1016/j.micron.2008.02.006 18555690

[B25] Menzel R Bachmann T Wesch W Physical sputtering of III-V-semiconductors with a focused Ga^+^−beam Nucl Instrum Methods Phys Res Sect B-Beam Interact Mater Atoms 1999 148 450 453 10.1016/S0168-583X(98)00861-1

[B26] Herrera M Ramasse QM Morgan DG Gonzalez D Pizarro J Yanez A Galindo P Garcia R Du MH Zhang SB Hopkinson M Browning ND Atomic scale high-angle annular dark field STEM analysis of the N configuration in dilute nitrides of GaAs Phys Rev B 2009 80 125211

[B27] Grillo V Carlino E Glas F Influence of the static atomic displacement on atomic resolution Z-contrast imaging Phys Rev B 2008 77 054103

[B28] Jia BY Yu ZY Liu YM Han LH Yao WJ Feng H Ye H Electronic structures of stacked layers quantum dots: influence of the non-perfect alignment and the applied electric field Chin Phys B 2011 20 027302 10.1088/1674-1056/20/2/027302

[B29] Nowak MP Szafran B Peeters FM Manipulation of two-electron states by the electric field in stacked self-assembled dots J Phys-Condes Matter 2008 20 395225 10.1088/0953-8984/20/39/395225

[B30] Springholz G Three-dimensional stacking of self-assembled quantum dots in multilayer structures C R Phys 2005 6 89 103 10.1016/j.crhy.2004.11.001

[B31] Kunert R Scholla E Strain-controlled correlation effects in self-assembled quantum dot stacks Appl Phys Lett 2006 89 153103 10.1063/1.2354476

